# Impact of biological and non-biological treatment on hematological indices in patients with ankylosing spondylitis and psoriatic arthritis

**DOI:** 10.1186/s43166-023-00174-0

**Published:** 2023-03-01

**Authors:** Fatma Mohammed Aboud, Salwa Galal, Menna Allah Zakaria Abou Elwafa, ALshymaa Mohammed Farouk

**Affiliations:** 1 l Medicine and Rheumatology, Faculty of Medicine, Ain Shams University, Cairo, Egypt; 2 Armed Forces College of Medicine, Cairo, Egypt; 3 Medicine, Rheumatology and Rehabilitation Department, Faculty of Medicine Ain Shams University, Cairo, Egypt; 4 Pathology, Faculty of Medicine, Ain Shams University, Cairo, Egypt

## Abstract

**Background:**

Blood dyscrasias are common in patients with rheumatic diseases, as bone marrow and blood cells can be targets for autoimmune processes. This in addition to the potentially adverse effect of the disease-modifying anti-rheumatic drugs used for the treatment of inflammatory arthritis as in psoriatic arthritis (PsA) and ankylosing spondylitis (AS) on blood counts.

**Aim of this study:**

The aim of this study is to analyze the effect of biologic therapy on complete blood cell parameters, derived ratios, and cell volume indices in Egyptian patients with ankylosing spondylitis and psoriatic arthritis.

**Results:**

One hundred and twenty Egyptian patients had been included, 60 have ankylosing spondylitis (AS) and 60 have psoriatic arthritis (PSA). On comparing the blood indices between the biologics and non-biologics groups of PSA patients, there was a statistically highly significant reduction in red cell distribution width (RDW%) at the biologics group than non-biologics (*p* < 0.006), where there was a statistically highly significant increase in Hb (hemoglobin)/RDW ratio and Hb/platelets ratio at the biologics group than non-biologics (*p* < 0.005).

**Conclusion:**

As a result, biologic drugs used in rheumatology practice may have some effects on hematological parameters. In our study, no major negative effects on hematological parameters were observed in patients with AS and PsA who received Secukinumab, Adalimumab-atto, or Golimumab biologic therapy. However, the changes in the hematological indices correlates with their potent anti-inflammatory action in rheumatic patients.

## Background

Autoimmune diseases are immune-mediated disorders affecting joints, kidneys, and others that usually alternate activity with quiescent stages [1]. These inflammatory disorders are characterised by, increased production of proinflammatory cytokines [2]. Which include tumor necrosis factor alpha (TNFα), interleukin-17 (IL-17), and interleukin (IL-6) [3]. Biologic therapy gives patients chance to improve their life by controlling the disease. Biologic therapy includes many drugs and biosimilars of different mechanisms. However, many patients showed minimal improvement while receiving biologics [4].

The use of anti-TNF agents has been also associated with laboratory abnormalities: hematological dyscrasias such as aplastic anemia, pancytopenia, and neutropenia have been rarely described, while it is more frequent the occurrence of non-organ specific auto-antibodies. However, related clinical autoimmune syndromes are rare and mostly reversible after anti-TNF treatment withdrawal [3]. It was found that neutropenia in patients treated with disease-modifying anti-rheumatic drugs occurs more in those with previous history of neutropenia. Seventy-four percent of patients developed neutropenia within 2 weeks of the treatment while the lymphocytes, monocytes, and basophils were increased. So, complete blood picture should be monitored after 1 month and every 3 to 6 months of biologic therapy [5]. Non-biologic disease modifying anti-rheumatic drugs (DMARDs) may also cause neutropenia and thrombocytopenia need regular monitoring as well [6].

In autoimmune rheumatic diseases, inflammatory markers and white blood cell count (WBC) can be used in assessing the disease activity. Recent studies suggested that the neutrophil/lymphocyte ratio (NLR), platelet/lymphocyte ratio (PLR), and RDW may be considered as markers of autoimmune rheumatic diseases (ARDs). The inflammation in ARDs leads to changes in the count, morphology, and sizes of blood cells. The blood cells indices could reflect the severity of inflammatory response in autoimmune diseases [7]. The complete blood picture and blood cell indices are cheap and easy tests that reflect the inflammatory status and the disease activity of ankylosing spondylitis (AS). All these indices could be affected with the use of DMARDs as well as biologics as their therapeutic role is to decrease inflammatory process in rheumatic diseases [8]. To our knowledge, there is no independent comparative retrospective study of this design has been published previously.

### Aim of the study

To analyze the impact of biological and non-biological treatment on hematological indices in patients with ankylosing spondylitis and psoriatic arthritis.

### Patients and methods

This cross-sectional study included 120 Egyptian patients, 60 with ankylosing spondylitis diagnosed according to the Assessment of Spondyloarthritis International Society (ASAS criteria) [9] and 60 psoriatic arthritis patients fulfilled the Classification criteria for the diagnosis of Psoriatic Arthritis (CASPAR) [10].

Patients recruited randomly from the outpatient clinics of Ain Shams University hospitals over 6 months. Patients with hematologic diseases, malignancy, chronic renal or liver disease and other autoimmune diseases were excluded. The study was approved from the Ethical Committee of Scientific Research, Faculty of Medicine, Ain Shams University. An informed consent was given by all participants. Full medical history was taken and detailed drug history including drug duration and type of DMARDs received by patients. General and musculoskeletal examination and complete blood picture were done to all patients. Different blood indices were assessed including WBCs, neutrophil and lymphocyte counts, red blood cell (RBCs) count, hemoglobin(Hb), hematocrit (HCT), mean corpuscular volume (MCV), mean corpuscular hemoglobin (MCH), red cell distribution width (RDW), platelets (PLTs), mean platelet volume (MPV), and platelet distribution width (PDW). Patients were divided according to therapy received into 2 groups: group I—patients on csDMARDs and group II—patients on biologic DMARDs. Comparison between both groups was done regarding the WBCs, RBCs, and platelet indices. Then, the patients on biologic therapy subdivided to groups according to the type of biologic drug.

#### Statistical analysis

Collected data were tabulated and statistically analysed using the statistical package for social sciences (SPSS) version 17.0. Variables were presented as frequencies and percentages, mean ± standard deviation and range. A comparison was done using chi-square and Mann–Whitney *U* tests. *P* value 0.05 was considered significant.

Hemoglobin/platelet ratio, RDW/PLT ratio, RBCs/PLT ratio, Hb/RDW ratio, neutrophil/lymphocyte ratio, and PLR:platelet/lymphocyte ratio.

## Results

The study included 120 Egyptian patients, 60 have ankylosing spondylitis (AS) and 60 have psoriatic arthritis (PSA). The PSA patients mean age was 44.7 ± 12.18 years. Twenty-two males and 38 females. The mean disease duration was 10.75 ± 6.32 years. Forty-three (71.67%) patients tested positive HLA B-27. The range and mean of blood indices, ESR, and CRP are shown in Table 1. 40 (66.67%) of these patients are on biological treatment in the form of anti-TNF, anti-TNF biosimilar, and anti-IL-17.

**Table 1:** Descriptive data of PsA patients

PSA				
Age (years)	Range	20	–	70
	Mean ± SD	44.750	±	12.187
DD (years)	Range	1	–	25
	Mean ± SD	10.750	±	6.316
		*N*		%
Sex	Male	22		36.67
	Female	38		63.33
WBCS	Range	3.6	–	13.3
	Mean ± SD	6.558	±	2.131
Neutrophils	Range	1.9	–	8.9
	Mean ± SD	4.148	±	1.580
Lymphocytes	Range	0.8	–	3.25
	Mean ± SD	1.993	±	0.600
HCT	Range	34	–	40.1
	Mean ± SD	37.088	±	1.567
RBCs	Range	4.2	–	6.2
	Mean ± SD	4.793	±	0.532
Hb	Range	11.4	–	15.2
	Mean ± SD	12.600	±	1.102
MCV	Range	64	–	100
	Mean ± SD	80.235	±	7.156
MCH	Range	12.7	–	30.3
	Mean ± SD	24.933	±	3.973
RDW%	Range	11.1	–	18.3
	Mean ± SD	13.655	±	2.015
PLT	Range	145	–	410
	Mean ± SD	247.833	±	72.786
MPV/fl	Range	7.1	–	12
	Mean ± SD	8.675	±	1.027
PDW	Range	7.9	–	18.8
	Mean ± SD	11.945	±	2.513
Hb/PLT ratio	Range	0.03	–	0.1
	Mean ± SD	0.055	±	0.017
RDW%/PLT ratio	Range	0.03	–	0.11
	Mean ± SD	0.059	±	0.018
RBCs/PLT ratio	Range	0.01	–	0.03
	Mean ± SD	0.021	±	0.006
Hb/RDW% ratio	Range	0.63	–	1.28
	Mean ± SD	0.941	±	0.153
Neutrophil/lymphocyte ratio	Range	1.36	–	3
	Mean ± SD	2.091	±	0.491
PLT/lymphocyte ratio	Range	60.42	–	300.77
	Mean ± SD	133.302	±	48.824
ESR	Range	8	–	180
	Mean ± SD	60.475	±	37.728
CRP	Range	2	–	42
	Mean ± SD	14.667	±	7.097
HLA B27	Positive	43		71.67
	Negative	17		28.33
SSZ	Yes	35		58.33
	No	25		41.67
CS	Yes	10		16.67
	No	50		83.33
MTX	Yes	47		78.33
	No	13		21.67
Biologics	No	20		33.33
	Golimumab	12		20.00
	Secukinumab	18		30.00
	Adalimumab-atto	10		16.67
Biologics	Non-biologics	20		33.33
	Biologics	40		66.67
Duration biologics (months)	Range	3	–	24
	Mean ± SD	8.100	±	4.361

*PsA* psoriatic arthritis, *DD* disease duration, *WBCs* white blood cells, *HCT* hematocrit, *RBCs* red blood cells, *HB* hemoglobin, *MCV* mean corpuscular volume, *MCH* mean corpuscular hemoglobin, *RDW* red cell distribution width, *PLT* platelet, *MPV* mean platelet volume, *PDW* platelet distribution width, *ESR* erythrocyte sedimentation rate, *CRP* C-reactive protein, *SSZ* sulfasalazine, *MTX* methotrexate, *SD* standard deviation

The AS patients mean age was 39.600 ± 12.048 years. Forty-one males and 19 females. The mean disease duration was 7.250 ± 5.634 years. 37 (61.67%) patients tested positive HLA B-27. The average and mean of blood indices all shown in Table 2. 28 (46.67%) of these patients are on biological treatment in the form of anti-TNF and anti-IL-17.

**Table 2:** Descriptive data of AS patients

AS				
Age (years)	Range	19	–	67
	Mean ± SD	39.600	±	12.048
DD (years)	Range	0.5	–	29
	Mean ± SD	7.250	±	5.634
		*N*		%
Sex	Male	41		68.33
	Female	19		31.67
WBCS	Range	4	–	12
	Mean ± SD	6.853	±	1.737
Neutrophils	Range	2.2	–	8.7
	Mean ± SD	3.988	±	1.311
Lymphocytes	Range	1.1	–	4
	Mean ± SD	2.443	±	0.792
HCT	Range	33	–	45
	Mean ± SD	38.140	±	3.213
RBCs	Range	3.9	–	7
	Mean ± SD	4.915	±	0.644
Hb	Range	11	–	15.6
	Mean ± SD	12.978	±	1.639
MCV	Range	57.1	–	93.4
	Mean ± SD	78.268	±	7.214
MCH	Range	14.2	–	30.7
	Mean ± SD	25.397	±	3.914
RDW%	Range	10	–	16.8
	Mean ± SD	13.232	±	1.600
PLT	Range	166	–	692
	Mean ± SD	309.650	±	121.259
MPV/fl	Range	5.7	–	19.6
	Mean ± SD	9.695	±	2.322
PDW	Range	8.1	–	18.8
	Mean ± SD	11.457	±	2.739
Hb/PLT ratio	Range	0.02	–	0.09
	Mean ± SD	0.048	±	0.018
RDW%/PLT ratio	Range	0.02	–	0.08
	Mean ± SD	0.048	±	0.016
RBCs/PLT ratio	Range	0.01	–	0.03
	Mean ± SD	0.018	±	0.007
Hb/RDW% ratio	Range	0.71	–	1.32
	Mean ± SD	0.997	±	0.179
Neutrophil/lymphocyte ratio	Range	0.96	–	6.45
	Mean ± SD	1.786	±	0.904
PLT/lymphocyte ratio	Range	50.3	–	259.09
	Mean ± SD	139.872	±	61.929
ESR	Range	5	–	100
	Mean ± SD	38.383	±	24.654
CRP	Range	2	–	30
	Mean ± SD	9.950	±	7.819
HLA B27	Positive	37		61.67
	Negative	23		38.33
SSZ	Yes	44		73.33
	No	16		26.67
CS	Yes	24		40.00
	No	36		60.00
MTX	Yes	44		73.33
	No	16		26.67
NSAIDs	Yes	58		96.67
	No	2		3.33
Biologics	No	32		53.33
	Golimumab	10		16.67
	Secukinumab	18		30.00
Biologics	Non-biologics	32		53.33
	Biologics	28		46.67
Duration biologics (months)	Range	3	–	15
	Mean ± SD	6.857	±	3.251

*AS* ankylosing spondylitis, *DD* disease duration, *WBCs* white blood cells, *HCT* hematocrit, *RBCs* red blood cells, *HB* hemoglobin, *MCV* mean corpuscular volume, *MCH* mean corpuscular hemoglobin, *RDW* red cell distribution width, *PLT* platelet, *MPV* mean platelet volume, *PDW* platelet distribution width, *ESR* erythrocyte sedimentation rate, *CRP* C-reactive protein, *SSZ* sulfasalazine, *MTX* methotrexate, *SD* standard deviation

On comparing the blood indices between the biologics and non-biologics groups of PSA patients, there was a statistically highly significant reduction in red cell distribution width (RDW%) at the biologics group than non-biologics (*p* < 0.006), where there was a statistically highly significant increase in Hb/RDW ratio and Hb/platelets ratio at the biologics group than non-biologics (*p* < 0.005). There was an increase in the hemoglobin (Hb), WBCs count (neutrophils and lymphocytes) and platelets distribution width, as well, in the biologics group than non-biologics group, however not statistically significant. There was a decrease in the RBCs, and platelets count in the biologics group than non-biologics, although not statistically significant (Table 3).

**Table 3:** Comparison between PSA patients with and without biologic therapy as regards WBCs, RBCs, platelets indices

PSA	Biologics						*t* test	
	Non-biologics			Biologics			*t*	*P* value
	Mean	±	SD	Mean	±	SD		
WBCS	6.135	±	2.029	6.770	±	2.174	− 1.090	0.280
Neutrophils	3.815	±	1.521	4.315	±	1.601	− 1.159	0.251
Lymphocytes	1.858	±	0.538	2.061	±	0.624	− 1.245	0.218
HCT	36.590	±	1.836	37.338	±	1.372	− 1.773	0.081
RBCs	4.895	±	0.669	4.742	±	0.449	1.052	0.297
Hb	12.380	±	1.010	12.710	±	1.142	− 1.095	0.278
MCV	79.830	±	10.495	80.438	±	4.866	− 0.308	0.759
MCH	25.350	±	4.261	24.725	±	3.860	0.571	0.570
RDW%	14.640	±	2.205	13.163	±	1.741	2.832	0.006*
PLT	265.250	±	79.122	239.125	±	68.786	1.319	0.192
MPV/fl	8.605	±	0.984	8.710	±	1.058	Gve3	0.712
PDW	11.325	±	2.085	12.255	±	2.673	− 1.361	0.179
Hb/PLT ratio	0.049	±	0.014	0.058	±	0.018	− 1.885	0.064
RDW%/PLT ratio	0.058	±	0.016	0.060	±	0.019	− 0.359	0.721
RBCs/PLT ratio	0.020	±	0.005	0.021	±	0.006	− 0.770	0.444
Hb/RDW% ratio	0.864	±	0.156	0.979	±	0.138	− 2.914	0.005*
Neutrophil/lymphocyte ratio	2.058	±	0.500	2.107	±	0.492	− 0.366	0.716
PLT/lymphocyte ratio	150.143	±	51.290	124.882	±	45.896	1.933	0.058

*PsA* psoriatic arthritis, *DD* disease duration, *WBCs* white blood cells, *HCT* hematocrit, *RBCs* red blood cells, *HB* hemoglobin, *MCV* mean corpuscular volume, *MCH* mean corpuscular hemoglobin, *RDW* red cell distribution width, *PLT* platelet, *MPV* mean platelet volume, *PDW* platelet distribution width, *SD* standard deviation. Level of significance: *P* ≤ .0.05: significant, *P* < 0.005: highly significant

On comparing the blood indices between the biologics and non-biologics groups of AS patients, there was a statistically highly significant reduction in red cell distribution width (RDW%) at the biologics group than non-biologics (*p* < 0.001), where there was a statistically highly significant increase in Hb/RDW ratio at the biologics group than non-biologics (*p* < 0.001). There was a statistically insignificant increase in the PLT/Lymphocyte ratio, in the biologics group than non-biologics group. There was a decrease in the WBCs count (mainly lymphocytes) in the biologics group than non-biologics, although not statistically significant (Table 4).

**Table 4:** Comparison between AS patients with and without biologic therapy as regards WBCs, RBCs, and platelets indices

AS	Biologics						*t* test	
	Non-biologics			Biologics				
	Mean	±	SD	Mean	±	SD	*t*	*P* value
WBCS	7.150	±	1.375	6.514	±	2.049	1.43	0.159
Neutrophils	4.116	±	0.997	3.843	±	1.605	0.80	0.426
Lymphocytes	2.616	±	0.809	2.246	±	0.737	1.84	0.071
HCT	37.713	±	3.451	38.63	±	2.903	− 1.104	0.274
RBCs	4.832	±	0.617	5.011	±	0.672	− 1.074	0.287
Hb	12.750	±	1.527	13.24	±	1.749	− 1.157	0.252
MCV	78.072	±	6.004	78.49	±	8.498	− 0.224	0.824
MCH	25.509	±	3.880	25.27	±	4.020	0.237	0.814
RDW%	13.984	±	1.538	12.37	±	1.201	4.480	< 0.001
PLT	301.7	±	84.9	318.71	±	153.9	− 0.538	0.592
MPV/fl	9.491	±	1.31	9.929	±	3.12	− 0.726	0.471
PDW	11.409	±	2.693	11.511	±	2.84	− 0.142	0.888
Hb/PLT ratio	0.047	±	0.017	0.050	±	0.019	− 0.739	0.463
RDW%/PLT ratio	0.049	±	0.014	0.046	±	0.018	0.80	0.428
RBCs/PLT ratio	0.018	±	0.007	0.019	±	0.007	− 1.034	0.306
Hb/RDW% ratio	0.923	±	0.146	1.081	±	0.179	− 3.78	< 0.001
Neutrophil/lymphocyte ratio	1.733	±	0.749	1.847	±	1.065	− 0.48	0.630
PLT/lymphocyte ratio	129.91	±	57.23	151.26	±	66.09	− 1.34	0.185

*AS* ankylosing spondylitis, *DD* disease duration, *WBCs* white blood cells, *HCT* hematocrit, *RBCs* red blood cells, *HB* hemoglobin, *MCV* mean corpuscular volume, *MCH* mean corpuscular hemoglobin, *RDW* red cell distribution width, *PLT* platelet, *MPV* mean platelet volume, *PDW* platelet distribution width, *SD* standard deviation. Level of significance: *P* ≤ 0.05: significant, *P* < 0.005: highly significant

The PSA patients on anti-IL-17 were able to significantly increase the WBCs count and MCH level compared to patients on anti-TNF and anti-TNF biosimilar respectively (*p* < 0.04). While patients on anti-TNF biosimilar showed statistically significant increase in RDW%/PLT ratio compared to patients on anti-IL17 and anti-TNF (*p* < 0.023), the treatment with anti-TNF significantly increase PLT/lymphocyte ratio in comparison to other patients on anti-IL17 and anti-TNF biosimilar (*p* < 0.031) Table 5.

**Table 5:** Comparison between different types of biologics in PsA as regards WBCs, RBCs, and platelets indices

PSA	Biologics									ANOVA	
	Golimumab			Secukinumab			Adalimumab-atto				
	Mean	±	SD	Mean	±	SD	Mean	±	SD	*F*	*P* value
WBCS	5.725	±	2.149	7.678	±	2.158	6.390	±	1.667	3.507	0.040*
Neutrophils	3.533	±	1.719	4.922	±	1.534	4.160	±	1.212	3.067	0.059
Lymphocyte	1.875	±	0.658	2.219	±	0.648	2.000	±	0.514	1.171	0.321
HCT	36.717	±	1.067	37.433	±	1.120	37.910	±	1.869	2.286	0.116
RBCs	4.703	±	0.474	4.739	±	0.396	4.794	±	0.545	0.109	0.897
Hb	12.375	±	0.609	12.739	±	1.309	13.060	±	1.295	0.991	0.381
MCV	79.233	±	5.262	80.700	±	4.902	81.410	±	4.501	0.581	0.565
MCH	22.425	±	4.020	25.511	±	3.826	26.070	±	2.623	3.510	0.040*
RDW%	12.333	±	1.233	13.772	±	1.923	13.060	±	1.616	2.699	0.080
PLT	269.917	±	83.981	232.056	±	54.072	214.900	±	65.910	2.018	0.147
MPV/fl	8.450	±	0.639	9.139	±	1.176	8.250	±	1.023	3.083	0.058
PDW	11.483	±	1.503	12.839	±	3.397	12.130	±	2.213	0.938	0.401
Hb/PLT ratio	0.049	±	0.013	0.058	±	0.015	0.066	±	0.023	2.795	0.074
RDW%/PLT ratio	0.048	±	0.013	0.062	±	0.016	0.069	±	0.024	4.191	0.023*
RBCs/PLT ratio	0.020	±	0.007	0.021	±	0.005	0.023	±	0.008	0.579	0.565
Hb/RDW% ratio	1.012	±	0.107	0.939	±	0.148	1.011	±	0.144	1.375	0.266
Neutrophil/lymphocyte ratio	1.877	±	0.560	2.254	±	0.399	2.118	±	0.500	2.266	0.118
PLT/lymphocyte ratio	153.553	±	47.659	113.377	±	43.496	111.184	±	34.773	3.841	0.031*

*PsA* psoriatic arthritis, *DD* disease duration, *WBCs* white blood cells, *HCT* hematocrit, *RBCs* red blood cells, *HB* hemoglobin, *MCV* mean corpuscular volume, *MCH* mean corpuscular hemoglobin, *RDW* red cell distribution width, *PLT* platelet, *MPV* mean platelet volume, *PDW* platelet distribution width, *SD* standard deviation. Level of significance: *P* ≤ 0.05: significant, *P* < 0.005: highly significant

In comparing between different types of biologics in AS as regards WBCs, RBCs, platelets indices, it did not show significant changes as shown in Table 6.

**Table 6:** Comparison between different types of biologics in AS as regards WBCs, RBCs, and platelets indices

AS	Biologics						*t* test	
	Golimumab			Secukinumab			*t*	*P* value
	Mean	±	SD	Mean	±	SD		
WBCS	5.950	±	1.769	6.828	±	2.173	− 1.090	0.286
Neutrophils	3.420	±	1.227	4.078	±	1.769	− 1.041	0.308
Lymphocytes	2.140	±	0.655	2.306	±	0.790	− 0.563	0.579
HCT	39.290	±	2.983	38.261	±	2.876	0.895	0.379
RBCs	5.020	±	0.547	5.006	±	0.748	0.053	0.958
Hb	13.40	±	1.627	13.150	±	1.853	0.356	0.724
MCV	82.01	±	6.748	76.539	±	8.904	1.687	0.104
MCH	25.800	±	3.883	24.972	±	4.175	0.515	0.611
RDW%	12.470	±	0.970	12.317	±	1.335	0.318	0.753
PLT	286.5	±	127.8	336.6	±	167.4	− 0.82	0.419
MPV/fl	10.21	±	3.67	9.772	±	2.87	0.350	0.729
PDW	10.65	±	2.19	11.99	±	3.1	− 1.21	0.239
Hb/PLT ratio	0.053	±	0.02	0.048	±	0.02	0.614	0.545
RDW%/PLT ratio	0.050	±	0.02	0.044	±	0.02	0.841	0.408
RBCs/PLT ratio	0.020	±	0.01	0.019	±	0.01	0.419	0.679
Hb/RDW% ratio	1.084	±	0.18	1.079	±	0.19	0.063	0.950
Neutrophil/lymphocyte ratio	1.651	±	0.59	1.956	±	1.26	− 0.72	0.478
PLT/lymphocyte ratio	142.7	±	64.9	156.1	±	68.2	− 0.51	0.618

*AS* ankylosing spondylitis, *DD* disease duration, *WBCs* white blood cells, *HCT* hematocrit, *RBCs* red blood cells, *HB* hemoglobin, *MCV* mean corpuscular volume, *MCH* mean corpuscular hemoglobin, *RDW* red cell distribution width, *PLT* platelet, *MPV* mean platelet volume, *PDW* platelet distribution width, *SD* standard deviation. Level of significance: *P* ≤ 0.05: significant, *P* < 0.005: highly significant

There was a statistically significant correlation between the changes in the WBCs count mainly neutrophils and the duration of biologic therapy (*p* < 0.03). However negative correlation between the duration of biologic therapy and HCT, RBCs, Hb. MCV, RDW, PLT, MPV/fl, PDW, Hb/PLT ratio, RDW%/PLT ratio, RBCs/PLT ratio, Hb/RDW% ratio, neutrophil/lymphocyte ratio, PLT/lymphocyte ratio, but it was not statistically significant (Table 7).

**Table 7:** Correlation between the WBCs, RBCs and Platelets indices with duration of biologic therapy

PSA	Duration biologics (months)	
	*r*	*P* value
WBCS	0.337	0.033*
Neutrophils	0.313	0.049*
Lymphocytes	0.265	0.098
HCT	− 0.127	0.436
RBCs	− 0.034	0.833
Hb	− 0.112	0.490
MCV	− 0.028	0.865
MCH	0.105	0.520
RDW%	0.163	0.316
PLT	0.134	0.411
MPV/fl	− 0.155	0.341
PDW	− 0.072	0.658
Hb/PLT ratio	− 0.121	0.457
RDW%/PLT ratio	− 0.088	0.591
RBCs/PLT ratio	− 0.068	0.676
Hb/RDW% ratio	− 0.151	0.351
Neutrophil/lymphocyte ratio	0.128	0.430
PLT/lymphocyte ratio	− 0.105	0.520

*PsA* psoriatic arthritis, *DD* disease duration, *WBCs* white blood cells, *HCT* hematocrit, *RBCs* red blood cells, *HB* hemoglobin, *MCV* mean corpuscular volume, *MCH* mean corpuscular hemoglobin, *RDW* red cell distribution width, *PLT* platelet, *MPV* mean platelet volume, *PDW* platelet distribution width, *SD* standard deviation. Level of significance: *P* ≤ 0.05: significant, *P* < 0.005: highly significant

No significant correlation between the duration of biologic treatment in AS patients and the levels of blood indices as shown in Table 8.

**Table 8:** Correlation between the WBCs, RBCs, and Plptelets indices with duration of biologic therapy

AS	Duration biologics (months)	
	*r*	*P* value
WBCS	− 0.030	0.879
Neutrophils	− 0.041	0.834
Lymphocytes	− 0.091	0.643
HCT	0.007	0.973
RBCs	0.102	0.604
Hb	− 0.008	0.967
MCV	− 0.143	0.468
MCH	0.138	0.483
RDW%	0.109	0.581
PLT	− 0.050	0.799
MPV/fl	0.341	0.076
PWD	0.230	0.239
Hb/PLT ratio	0.018	0.928
RDW%/PLT ratio	0.021	0.914
RBCs/PLT ratio	0.133	0.501
Hb/RDW% ratio	− 0.057	0.773
Neutrophil/lymphocyte ratio	0.096	0.626
PLT/lymphocyte ratio	0.015	0.939

*AS* ankylosing spondylitis, *DD* disease duration, *WBCs* white blood cells, *Neut*. neutrophils, *HCT* hematocrit, *RBCs* red blood cells, *HB* hemoglobin, *MCV* mean corpuscular volume, *MCH* mean corpuscular hemoglobin, *RDW* red cell distribution width, *PLT* platelet, *MPV* mean platelet volume, *PDW* platelet distribution width, *Lympho* lymphocyte, *SD* standard deviation. Level of significance: *P* ≤ 0.05: Significant, *P* < 0.005: highly significant

## Discussion

Blood cell abnormalities are common in autoimmune rheumatic diseases [11]. In addition to the potentially adverse effect of the treatment therapy of inflammatory arthritis as in PSA and AS on blood counts.

White blood cells and platelets are involved in the inflammatory response in autoimmune diseases and malignancies. Neutrophils activate the antigen-presenting cells and platelets boost the leukocyte recruitment [12]. In this cross-sectional study, we performed a comprehensive analysis of different hematological indices, such as Hb/PLT ratio, RDW%/PLT ratio, RBCs/PLT ratio, Hb/RDW% ratio, neutrophil/lymphocyte ratio, and PLT/lymphocyte ratio, in 120 Egyptian patients, 60 have ankylosing spondylitis (AS) and 60 have psoriatic arthritis some receiving DMARDs and others on biologic therapy, to distinguish the different effects of biologic therapy on these indices.

Many studies suggested that treatment with biologic therapy leads to neutropenia [13–15]. However, in the current study, we had discovered that some TNF inhibitors studied here like Golimumab, TNF inhibitors biosimilar like Adalimumab-atto, and anti-IL-17 like Secukinumab used in treatment of PSA patients, may increase WBCs count above the non-biologics group, with no significant difference between each of them, this was in concordance with Pereckova, J et al. 2022 who reported the significant increase in the WBCs count in RA biologic group than healthy control group. A reported increase in neutrophils and lymphocytes count in the biologic group than non-biologic group although non-significant, it might reflect the role of anti-TNF in the growth of hematopoietic stem and progenitor cells and its suppression is associated with lymphoproliferative disorders and pathological abnormalities [16].

The RDW is a traditional hematologic index and used to estimate erythrocyte variability, and determine the type of anemia, it is also used as a novel index for inflammation, assessment of diseases activity and prognosis of various diseases including rheumatic diseases [17]. While the Hb/RDW ratio has a strong prognostic role in many diseases as it was mentioned that patients with a lower Hb/RDW ratio were more likely to have more comorbidities [18]. Corresponding to the data of this study, there was a significant decrease in the RDW in the biologically treated group than non- biologic treated group in PSA and AS patients as well, and this may refer to the improvement of the activity and anemia of chronic diseases this was in agreement with Moreno-Torres V et al. 2022, while the Hb/RDW ratio showed significant increase in biologic group than non-biologic group in PSA as well as AS patients, this could be attributed to the lower levels of RDW and improvement of prognosis and comorbidities associated with autoimmune rheumatic diseases [19]. However, other hematological indices did not show any significant differences between biologic and non-biologic groups in AS and PSA patients.

On comparing the effect of each biologic drug on the hematological parameters and indices, it showed no significant difference between different types of biologics in AS patients, while in PSA patients there was a significant difference in the WBCs count which showed significant increase in Secukinumab group than Adalimumab-atto and Golimumab groups, this was explained by Karataş A et al. 2022 [20] who concluded that no negative effects on hematological parameters were observed in patients with AS and PSA who received Secukinumab therapy. While the RDW/platelets ratio and platelets/lymphocytes ratio showed higher levels in Adalimumab-atto and Golimumab groups, respectively, than Secukinumab group. RDW/platelets ratio and platelets/lymphocytes are related to laboratory and clinical parameters of disease activity in rheumatic diseases. These indices may be used in identifying and following active patients. TNF-alpha is a key cytokine of the immune system that induces several chemokines, endothelial adhesion molecules (ICAM-1, VCAM-1 …), inflammatory cytokines and also enhances leukocyte recruitment and migration. Thus, it has a key role in systemic inflammation, all of these can explain the higher values of hematologic indices in anti-TNF patients [21].

An I, et al. [22] showed that the NLR was decreased in psoriatic arthritis patients treated with biologics irrespective to the type of the drug used. In our study, we observed that, unlike the above data, it showed, there was a positive correlation between WBCs/neutrophils count and the duration of biologic therapy. However, in AS patients there was no significant correlation between the duration of the biologic therapy and the hematological indices.

### Limitations of this study

Retrospective design of the study, being a single-center and small sample size; with small number of patients receiving biologic therapy. All were limitations of this study.

The small presented number of patients can explain some unusual findings like the relatively higher incidence of HLAB-27 positivity in PsA than AS patients and high ESR, which is also could be explained by the co-incidence of COVID-19 infection in 2 patients (who skipped one dose of Golimumab), and a resistant urinary infection in another patient, and blood tests were taken only once from each patient.

## Conclusion

Biologic drugs used in rheumatic diseases may affect the hematological parameters. In current study, no major negative effects on hematological parameters were observed in patients with AS and PsA who received Secukinumab, Adalimumab-atto, or Golimumab biologic therapy. However, the changes in the hematological indices correlate with their potent anti-inflammatory action in rheumatic patients.

## Data Availability

The data of the current study are available from the corresponding author on reasonable request.

## Abbreviations

PsA: Psoriatic arthritis
AS: Ankylosing spondylitis
DD: Disease duration
Neut: Neutrophils
Lymph: Lymphocytes
HCT: Hematocrit
RBCs: Red blood cell
Hb: Hemoglobin
MCV: Mean corpuscular volume
MCH: Mean corpuscular hemoglobin
RDW: Red cell distribution width
PLT: Platelets
MPV: Mean platelet volume
PDW: Distribution width platelets
Cs: Corticosteroids
MTX: Methotrexate
HCQ: Hydroquine
SSZ: Sulfasalazine
